# Comparison of inferred relatedness based on multilocus variable-number tandem-repeat analysis and whole genome sequencing of *Vibrio cholerae* O1

**DOI:** 10.1093/femsle/fnw116

**Published:** 2016-04-28

**Authors:** Mahamud-ur Rashid, Mathieu Almeida, Andrew S. Azman, Brianna R. Lindsay, David A. Sack, Rita R. Colwell, Anwar Huq, J. Glenn Morris, Munirul Alam, O. Colin Stine

**Affiliations:** 1 School of Medicine, University of Maryland, Baltimore, MD 21201, USA; 2 Department of Microbiology, International Centre for Diarrheal Disease Research, Mohakhali, 1212 Dhaka, Bangladesh; 3 Center for Bioinformatics and Computational Biology, University of Maryland, Paint Branch Road, College Park, MD 20742, USA; 4 Department of International Health, Johns Hopkins Bloomberg School of Public Health, North Wolfe Street, Baltimore, MD 21205, USA; 5 Merck & Co., Philadelphia, PA 19454, USA; 6 Maryland Pathogen Research Institute, College of Chemical and Life Sciences, University of Maryland, College Park, MD 20742, USA; 7 Emerging Pathogens Institute, University of Florida, 2055 Mowry Road, Gainesville, FL 32610, USA

**Keywords:** cholera, *Vibrio cholerae*, multilocus variable tandem-repeat analysis (MLVA), whole genome sequencing (WGS), recombination

## Abstract

*Vibrio cholerae* causes cholera, a severe diarrheal disease. Understanding the local genetic diversity and transmission of *V. cholerae* will improve our ability to control cholera. *Vibrio cholerae* isolates clustered in genetically related groups (clonal complexes, CC) by multilocus variable tandem-repeat analysis (MLVA) were compared by whole genome sequencing (WGS). Isolates in CC1 had been isolated from two geographical locations. Isolates in a second genetically distinct group, CC2, were isolated only at one location. Using WGS, CC1 isolates from both locations revealed, on average, 43.8 nucleotide differences, while those strains comprising CC2 averaged 19.7 differences. Strains from both MLVA-CCs had an average difference of 106.6. Thus, isolates comprising CC1 were more closely related (*P* < 10^−6^) to each other than to isolates in CC2. Within a MLVA-CC, after removing all paralogs, alternative alleles were found in all possible combinations on separate chromosomes indicative of recombination within the core genome. Including recombination did not affect the distinctiveness of the MLVA-CCs when measured by WGS. We found that WGS generally reflected the same genetic relatedness of isolates as MLVA, indicating that isolates from the same MLVA-CC shared a more recent common ancestor than isolates from the same location that clustered in a distinct MLVA-CC.

## Abstract (precis)

Genetic relatedness of *Vibrio cholerae* isolates from distinct locations was similar when measured by multilocus variable tandem-repeat analysis or whole genome sequencing.

## INTRODUCTION

*Vibrio cholerae* causes cholera, a severe diarrheal disease, with hundreds of thousands of cases recorded annually in Africa and Asia (Ali *et al.*^2015^). Understanding local transmission dynamics of *V. cholerae* should improve our ability to control this life-threatening disease and molecular methods offer insight complementary to traditional epidemiologic data analyses. Extensive genetic variation among cholera strains has been observed by multilocus variable-number tandem-repeat analysis (MLVA) (Kendall *et al.*^2010^). These repeats are short DNA sequence motifs that are repeated multiple times (2–25, with the number of repeats determining the allele number) at a specific locus. For *V. cholerae*, at least five tandem-repeat loci are highly polymorphic (Danin-Poleg *et al.*^2007^). MLVA of these loci has revealed a clustering of clinical isolates of *V*. *cholerae* from different geographic regions and different times of collection (Ghosh *et al.*^2008^; Stine *et al.*^2008^). Rashed *et al.* (2014) showed that, in two Bangladeshi communities, one group of related genotypes, a clonal complex (MLVA-CC), differentiated into 26 genotypes. Furthermore, in one of the communities, only isolates from this MLVA-CC had been isolated whereas in the other community, additional MLVA-CCs were identified during a year-long clinical and environmental surveillance. Isolates comprising a single MLVA-CC are hypothesized to have arisen from a common ancestor, with isolates of two separate MLVA-CCs assumed to represent independent genetic lineages.

Whole genome sequencing (WGS) offers a more precise identification of genetic lineages and, when combined with phylogenetic analysis, can be used to estimate migration patterns (over time and space) of isolates within lineages. Results from WGS of *V. cholerae* isolates have been interpreted as identifying three waves of migration across the globe over the past 50 years (Mutreja *et al*. 2011). The third wave interpreted by the authors to include the introduction of *V. cholerae* into Haiti by strains from Asia (Chin *et al.*^2011^). WGS of *V. cholerae* isolates from Kenya showed two distinct genetic lineages residing in the country for about 10 years (Kiiru *et al.*^2013^)—a qualitative conclusion drawn in a separate analysis employing MLVA (Mohamed *et al.*^2012^).

In two previous studies of *V. cholerae*, results obtained using MLVA and WGS were compared. The first compared inferred relationships between 66 *V. cholerae* isolates collected over a 38-year period from various geographic locations throughout the world. The authors found no clear evidence of any relationship among the MLVA genotypes and single nucleotide polymorphism profiles and concluded that MLVA is useful only for analysis of isolates collected within a shorter time frame or geographic scale (Lam *et al.*^2010^). In addition, a study of 38 isolates of *V. cholerae* from a single outbreak in northern India showed that MLVA discriminated isolates of the clades identified by WGS, but one small WGS clade contained isolates from three MLVA-CC (Abd El Ghany *et al.*^2014^). Neither study, however, systematically compared relatedness as measured by MLVA and by WGS.

In this study, strains of *V. cholerae*, comprising two clonal complexes, isolated from samples collected at two sites in Bangladesh located ca. 400 km apart (Rashed *et al.*^2014^), were examined using WGS analysis to determine whether isolates of the same MLVA clonal complex but from samples collected at different locations were more recently derived from the same common ancestor than isolates from different clonal complexes at a single location.

## METHODS AND MATERIALS

### Bacterial isolates

A total of 42 *V. cholerae* O1 isolates were selected for study based on their MLVA genotype (Rashed *et al.*^2014^). Isolates with the same MLVA genotype comprising CC1 were from Mathbaria (*n* = 2) and from Chhatak (*n* = 5). Isolates with different MLVA genotypes, but related by one or more successive changes of a single allele and also a member of CC1, from Mathbaria (*n* = 4) and from Chhatak (*n* = 6) were included. Isolates of the same genotype in CC2 from Mathbaria (*n* = 9), different MLVA genotypes from CC2 from Mathbaria (*n* = 5) and isolates representing three additional complexes, CC3 (*n* = 3), CC4 (*n* = 2), CC5 (*n* = 2), and four singleton strains (defined as unrelated to any of the other strains of this study at two or more loci) comprised the full set of genotypes included in this analysis.

### Genomic DNA preparation

Genomic DNA was extracted from 3.0 ml of cells harvested from overnight LB broth culture using alkaline lysis followed by phenol–chloroform extraction, as described elsewhere (Chowdhury *et al.*^2000^). The harvested DNA was stored at –20°C.

### Genome sequencing

Genome sequencing of the 42 *V. cholerae* O1 isolates was performed using DNA prepared for Illumina sequencing with the KAPA High Throughput Library Preparation Kit (Kapa Biosystems, Wilmington, MA, USA). DNA was fragmented with the Covaris E210. Libraries were prepared using a modified version of the with-bead protocol (Kapa Biosystems). The libraries were enriched and barcoded by ten cycles of PCR amplification with primers containing an index sequence of seven nucleotides in length. The libraries were sequenced on a 100-bp-paired-end run on an Illumina HiSeq2500 (Illumina, San Diego, CA, USA).

### Whole genome alignment and detection of single nucleotide variants

The quality of the 101-bp-end reads was confirmed using FastQC (http://www.bioinformatics.bbsrc.ac.uk/projects/fastqc). High quality reads were assembled with ‘Spades’ software (v.3.6.2) (Bankevich *et al.*^2012^), using the options ‘–careful’ to reduce the number of mis-assemblies and ‘–cov-cutoff auto’ to remove the potentially mis-assembled low coverage contigs. This package produces a ‘*de novo*’ assembly and is not dependent upon a previously published sequence for comparison. The advantage is that if any section of the genome is present in the assembled sequence and not in the reference sequence, that sequence section will be included in the assembly and analysis. The MLVA loci were omitted from the WGS analysis because the length of the alleles often exceeded the read length. Annotation was performed using the RAST server (Aziz *et al.*^2008^). The assembled annotated files have been deposited in Genbank (SAMN03839339 to SAMN03839380). Nucleotide variations were identified by comparing with *V. cholerae* O1 El Tor reference strain N16961 (NCBI accession numbers AE003852 and AE003853) using default settings in CSI Phylogeny 1.0a (http://cge.cbs.dtu.dk/services/CSIPhylogeny/, Kaas *et al.*^2014^). CSI Phylogeny uses Burrows-Wheeler Aligner, SAMtools, BEDTools, MUMmer and FastTree, in succession, to generate a tree of genetic relatedness based on high-quality single nucleotide variants (SNVs). The program used default values to call, filter and validate the SNVs and based on concatenated alignment of the high quality SNVs, constructed a maximum-likelihood SNV tree of genetic relatedness (Kaas *et al.*^2014^). The resulting tree was visualized using FigTree v1.4.1 (http://tree.bio.ed.ac.uk/software/figtree/). High-density SNV clusters and possible recombination sites were identified using a previously described method (Croucher *et al.*^2011^). Identical results were obtained using SMALT (http://www.sanger.ac.uk/resources/software/smalt/).

Recombination analyses: SNVs were defined as those variants found in one or more isolates. Unique variants were found as those in only one isolate. The core genome was defined as those genes present in all genomes of this analysis that did not have a paralog. PARSNP (v1.2) (Treangen *et al.*^2014^) was used to extract and align variable nucleotides from the core genome, using the parameter ‘–c’ to constrain the use of all input genomes. Gingr (v1.2) (Treangen *et al.*^2014^) was used to export a file of the aligned variants which was loaded into Splitstree (Huson and Bryant 2006) to determine networks.

### Statistical analysis

To understand how well MLVA-CC genotypes aligned with those obtained using WGS, pairwise SNV differences within and between clonal complexes were compared. A permutation test was used to determine whether a mean pairwise SNV difference within the clonal complexes was significantly smaller than the mean pairwise SNV difference between clonal complexes.

## RESULTS

A total of 42 genomes with known MLVA genotypes were sequenced, analyzed and annotated (Table 1). Relationships between genotypes within the MLVA-CC of this study have been published previously and are shown in Fig. S1, Supporting Information (Rashed *et al.*^2014^). Average quality score ranged from 37 to 39 out of 40 and average depth of reads exceeded 100 times across the entire genome when compared to the reference sequences (*V. cholerae* O1 N16961). The genome assemblies contained an average of 128 contigs per isolate (range, 80–313), with an average G + C content of 47.45% (46.96%–47.49%) and 4.05 Mb average genome size (3.95–4.28 Mb).

**Table 1.** Strains and source, MLVA genotype, clonal complex and WGS summary.

Strain ID	Location	MLVA genotye	MLVA clonal	Genome size	GC %	No. of	Variants	found
			complex	(bp)		contigs	uniquely	multiply
NHCC_011	Chhatak	9-4-14-9-17	C1	4043 234	47.49	105	5	5
NHCC_019	Chhatak	9-4-14-21-12	C1	4021 528	47.48	313	1	11
NHCC_042	Chhatak	9-4-14-21-17	C1	4047 248	47.48	111	0	6
NHCC_048	Chhatak	9-4-14-25-16	C1	4048 869	47.48	122	4	10
NHCC_05	Chhatak	9-4-14-14-16	C1	4071 087	47.5	118	6	4
NHCC_068	Chhatak	8-4-14-21-18	C1	4042 585	47.49	137	1	5
NHCC_078	Chhatak	9-4-14-23-18	C1	4041 484	47.49	152	19	8
NHCC_079	Chhatak	9-4-14-23-18	C1	4045 004	47.48	109	2	12
NHCC_080	Chhatak	9-4-14-23-18	C1	4040 457	47.49	125	2	12
NHCC_081	Chhatak	9-4-14-23-18	C1	4044 220	47.49	108	2	7
NHCC_083	Chhatak	9-4-14-23-18	C1	4040 830	47.48	103	36	9
EM_1706	Mathbaria	10-4-14-9-18	C1	3952 250	47.6	100		
NHCM_043	Mathbaria	9-4-14-22-18	C1	4064 275	47.46	203	1	11
NHCM_044	Mathbaria	9-4-14-23-18	C1	4040 600	47.5	147	2	12
NHCM_045	Mathbaria	10-4-14-23-18	C1	4043 932	47.48	109	0	10
NHCM_047	Mathbaria	9-4-14-23-18	C1	4048 862	47.48	125	1	12
NHCM_054	Mathbaria	9-4-14-17-18	C1	4044 549	47.48	90	2	6
EM_1688	Mathbaria	11-9-14-15-18	C2	4063 740	47.46	156	1	7
EM_1690	Mathbaria	11-9-14-15-18	C2	4063 337	47.45	90	0	10
EM_1690A	Mathbaria	11-9-14-15-18	C2	3969 556	47.48	113	0	2
NHCM_012	Mathbaria	11-9-14-15-18	C2	4066 680	47.46	102	0	3
NHCM_013	Mathbaria	11-9-14-15-18	C2	4064 645	47.45	165	1	7
NHCM_016A	Mathbaria	11-9-14-15-18	C2	4062 074	47.45	100	2	6
NHCM_017	Mathbaria	11-9-14-15-18	C2	4280 674	46.96	247	0	5
NHCM_029	Mathbaria	11-9-14-15-18	C2	4065 570	47.45	98	1	6
NHCM_033	Mathbaria	11-9-14-15-19	C2	4098 822	47.46	96	1	4
NHCM_037	Mathbaria	12-9-14-15-18	C2	4061 882	47.46	115	0	10
NHCM_04	Mathbaria	11-9-14-15-18	C2	4059 738	47.46	104	0	3
NHCM_048	Mathbaria	11-9-7-?-16	C2	3964 912	47.48	131	2	3
NHCM_053	Mathbaria	11-9-14-15-16	C2	4062 854	47.45	87	1	3
NHCM_06	Mathbaria	11-9-14-15-17	C2	4074 374	47.44	180	1	3
EM_1542	Mathbaria	10-7-14-14-15	C3	4064 555	47.45	155		
EM_1543	Mathbaria	10-7-14-14-16	C3	4071 079	47.45	108		
NHCM_01	Mathbaria	11-7-14-14-15	C3	4067 119	47.45	155		
EM_1626	Mathbaria	9-4-14-14-16	C4	4065 819	47.45	99		
EM_1652A	Mathbaria	9-4-14-11-16	C4	4059 450	47.47	80		
NHCM_02	Mathbaria	11-8-14-13-19	C5	4065 626	47.45	103		
NHCM_03	Mathbaria	11-8-14-14-19	C5	4069 444	47.45	100		
EC_51	Chhatak	10-8-14-17-18	Singleton	4077 853	47.38	137		
NHCC_021	Chhatak	9-4-6-?-11	Singleton	4051 529	47.48	113		
NHCC_04	Chhatak	11-8-7-?-17	Singleton	4048 335	47.5	158		
EM_1654	Mathbaria	9-9-14-19-16	Singleton	4060 969	47.47	101		

Variable nucleotides were found among isolates within the MLVA-CCs using CSI-Phylogeny (http://cge.cbs.dtu.dk/services/CSIPhylogeny/, Kaas *et al.*^2014^), with a total of 553 variable nucleotides identified among the 42 isolates, compared to *V. cholerae* N16961 reference sequences. Pairwise nucleotide differences, defined as two genome sequences showing alternative nucleotides at the same locus, ranged from 11 to 251 for the 42 isolates. The 18 isolates comprising the CC1 established by MLVA showed pairwise nucleotide differences based on WGS ranging from 11 to 149, with an average of 43.8 and standard deviation of 30.0. Isolates comprising CC2 had pairwise nucleotide differences ranging from 11 to 36, with an average of 19.7 and standard deviation of 4.8 nucleotide differences. Pairwise nucleotide differences for CC3, CC4 and CC5, isolates ranged from 16 to 25, 88 and 16, respectively.

**Figure 1.**
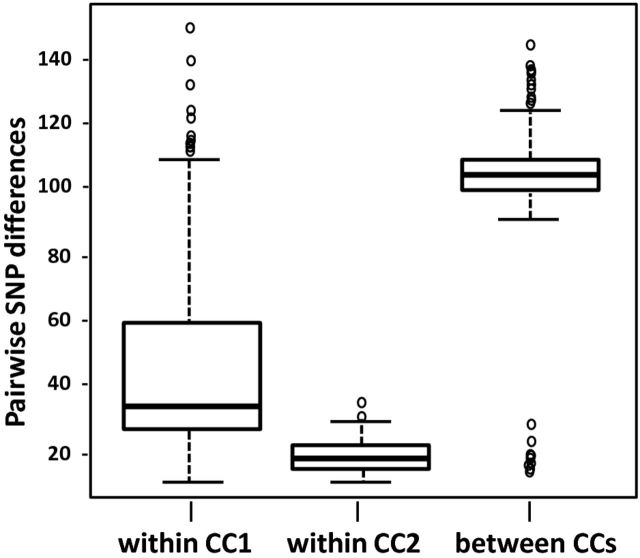
Variable nucleotides within and between clonal complexes 1 and 2. Shown are all possible pairs of the number of variable nucleotide differences and plotted for CC1, CC2 and pairs between CC1 and CC2.

Pairwise nucleotide differences for isolates comprising CC1 and CC2 ranged from 15 to 144, with an average of 106.6 and standard deviation of 10.1. The SNV difference distributions of all pairs, both within and between MLVA-CCs 1 and 2 are shown graphically in Fig. 1. Mean pairwise SNV differences for isolates comprising CC1 and CC2 were significantly smaller than mean pairwise differences between the clonal complexes (43.8 versus 106.6, permutation test *P* < 10^−6^).

**Figure 2.**
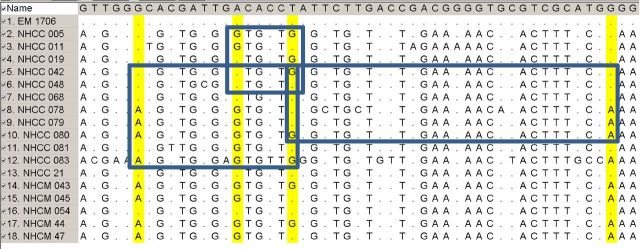
Demonstration of recombination. The displayed genetic sequence contains 51 of 104 variable nucleotides from isolates in MLVA CC1 determined by PARSNP. The dots indicate the nucleotide is the same as that in the top row. The highlighted columns mark selected loci where there are two alleles and the boxes include chromosomes that have all four combinations of the two alleles.

SNVs in each clonal complex were examined using PARSNP (v1.2) (Treangen *et al.*^2014^) as follows. The number of unique variants (present only in a single isolate) and the number of variants in more than one isolate for each isolate in clonal complex 1 and 2 was determined (Table 1). For 26 of 30 isolates, the number of unique variants is smaller than number of variants in multiple isolates. These results suggest that the stems of the phylogenetic tree should have a longer length than the terminal branches of a standard bifurcating tree. This was not the case (Fig. S2, Supporting Information). In the bifurcating tree, the terminal branches (the length of which is proportional to the distance or number of nucleotide changes) are longer than the shared length along the stems. The nucleotide changes within clonal complex 1 (Fig. 2) showed that at three selected loci the alternative alleles occur in all possible combinations (e.g. AA, AG, GA and GG) on separate chromosomes. This observation is consistent with recombination. To determine that this was not because of mis-assembly of paralogs, all paralogs were removed and only the remaining 2543 genes were analyzed. An alternative explanation that these parallel changes were from mutation was calculated to be less than 1.6 × 10^−13^.

**Figure 3.**
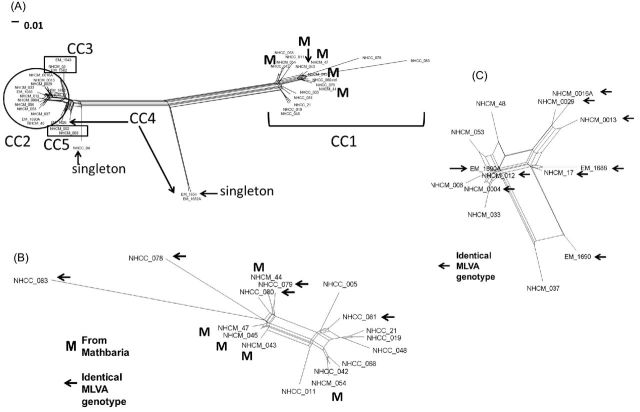
Phylogenetic relatedness of isolates of *V. cholerae* by WGS. Variants in the core genome of the assembled *V. cholerae* were defined and extracted using PARSNP and Gingr, as described in the method section, and loaded in Splitstree (v4_14_2) for analysis and display. The branch lengths are proportional to the number of variable nucleotides. (**A)** A total of 42 isolates and the reference strain N16961. The genomes of isolates from MLVA CC1, CC2, CC3, CC4 and CC5 are marked. **(B)** Isolates from CC1. The arrows identify those isolates with the identical MLVA genotype. ‘M’ marks those isolates from Mathbaria. **(C)** Isolates from CC2. The arrows identify those isolates with identical MLVA genotypes.

The presence of recombination among the chromosomes required network analysis of genetic relatedness. As shown in Fig. 3A, the network based on WGS separated CC1 from CC2, while CC3 and CC5 were closely related to CC2. Isolates from CC4 and one isolate of CC1 were determined to be genetically separate by WGS from their MLVA-CC. Isolates with an identical MLVA genotype and clustered in either CC1 (Fig. 3B) or CC2 (Fig. 3C) did not form a cluster in the respective networks. In addition, isolates in CC1 from Mathbaria did not comprise a cluster.

## DISCUSSION

Isolates collected at two sites in Bangladesh located 400 km apart geographically but comprising the same MLVA-derived clonal complex (CC1) were more closely related to each other, based on WGS, than to isolates from a different MLVA-CC (CC2). MLVA and WGS would be expected to provide results reflecting the same genetic history, since both methods trace genetic relatedness based on distinct loci. On the other hand, circumstances will dictate whether the two measures reflect the same history since WGS genotypes are defined by a larger number of loci. Our observation of recombination in the core genome increases the complexity of tracking strains within a relatively small spatial and temporal scale, while variations in MLVA yield an interpretable pattern, an advantage for MLVA.

Distinct MLVA clonal complexes often, but not necessarily, identify distinct genetic lineages. CC1, CC2, CC3 and CC5, for example, were genetically distinguishable by MLVA and WGS. Isolates from CC2, CC3 and CC5 were very closely related (≤48 bp) according to WGS and more closely related than many isolates from CC1 were related to other members of CC1. It should be noted that CC3 and CC5 each contains only two genotypes and comprised two or three isolates. Whether these become distinct lineages or remain a single lineage may be resolved by more intense sampling or in future collections.

Three isolates showed non-concurrence between MLVA and WGS. CC4 did not form a coherent group, when assessed by WGS. This MLVA-CC had only two genotypes and consisted of two isolates. It also demonstrated a convergence of MLVA genotypes, since one of the genotypes was identical to a CC1 genotype from Chhatak, while WGS identified this genotype as unrelated (91–128 SNVs distant) to CC1. A second example of non-coherence is EM_1706, an outlier in CC1 (range 114–159 SNV to other members of CC1, while other members had an 82 SNV pairwise maximum). Given the limited number of loci and alleles measured by MLVA, some convergence is bound to occur and may explain the lack of congruence seen previously between isolates over large time scales (Lam *et al.*^2010^). The consistency between MLVA-CC and WGS clades in our study is different from a previous report which found that both one WGS clades contained isolates from three MLVA_CCs (Abd El Ghany *et al.*^2014^). However, their result is limited by the small sample size in one WGS clade (*n* = 3). However, MLVA is easy to do, reagents are readily available and it is discriminative on a short time scale (outbreaks).

The core genome of *V. cholerae* recombines, contrary to the null hypothesis that the core genome does not recombine. Our identification of recombination events is concluded from the observation of two alleles at two loci with those alleles occurring in all four combinations (AA, GG, AG and GA). The presence of all four combinations of alleles at linked loci is the classic demonstration of recombination because two independent mutations at the nucleotide on alternative chromosomes in the same population are extremely rare. An exception is when the rate of mutation is very high as in the slip mispairing mutations in tandem repeats. For *V. cholerae*, recombination in the core genome is consistent with the presence of a molecular mechanism (Seitz and Blokesch 2013; Antonova and Hammer 2015) and recombination between serogroups (Blokesch and Schoolnik 2007; Gonzalez-Fraga *et al.*^2008^). The barrier to recognizing recombination events in the core genome has been an absence of genetic variation between isolates. Our sample is unusual in a way that many of our fully sequenced isolates were isolated from samples collected in the same geographic location at the same time. Hence, proximity serves as the prerequisite for recombination.

Recombination increases the difficulty of tracing strains on small spatial and temporal scales. If mutation were the only mechanism of change, then tracing strains would depend solely on distance along the bifurcating tree. However, once recombination intervenes, the appropriate path around the network becomes ambiguous. For MLVA, it is unlikely that two MLVA loci will be involved in a single recombination event because they are at least 115 kb distant and an event involving only one locus will appear to be a mutation.

The presence of the CC1 genetic lineage in both Mathbaria and Chhatak indicates a geographically and ecologically broad distribution for this genetic lineage in the areas of Bangladesh included in this study. Ecological and/or immunological selection patterns should prove useful in explaining this pattern of occurrence (Jutla *et al.*^2013^).

In our study, MLVA and WGS generally reflect the same genetic relatedness of *V. cholerae* isolates; MLVA, with sufficient numbers and recognizing the potential for outliers, can address hypotheses concerning migration of strain genotypes across geographic regions.

## Supplementary Material

Supplementary Data
