# Supplementary figures and images for: Comparison of inferred relatedness based on multilocus variable-number tandem-repeat analysis and whole genome sequencing of Vibrio cholerae O1
Source: FEMS Microbiol Lett. 2016 Apr 28;363(12):fnw116. doi: 10.1093/femsle/fnw116 (PMC4876684; doi:10.1093/femsle/fnw116)

## Slide 1
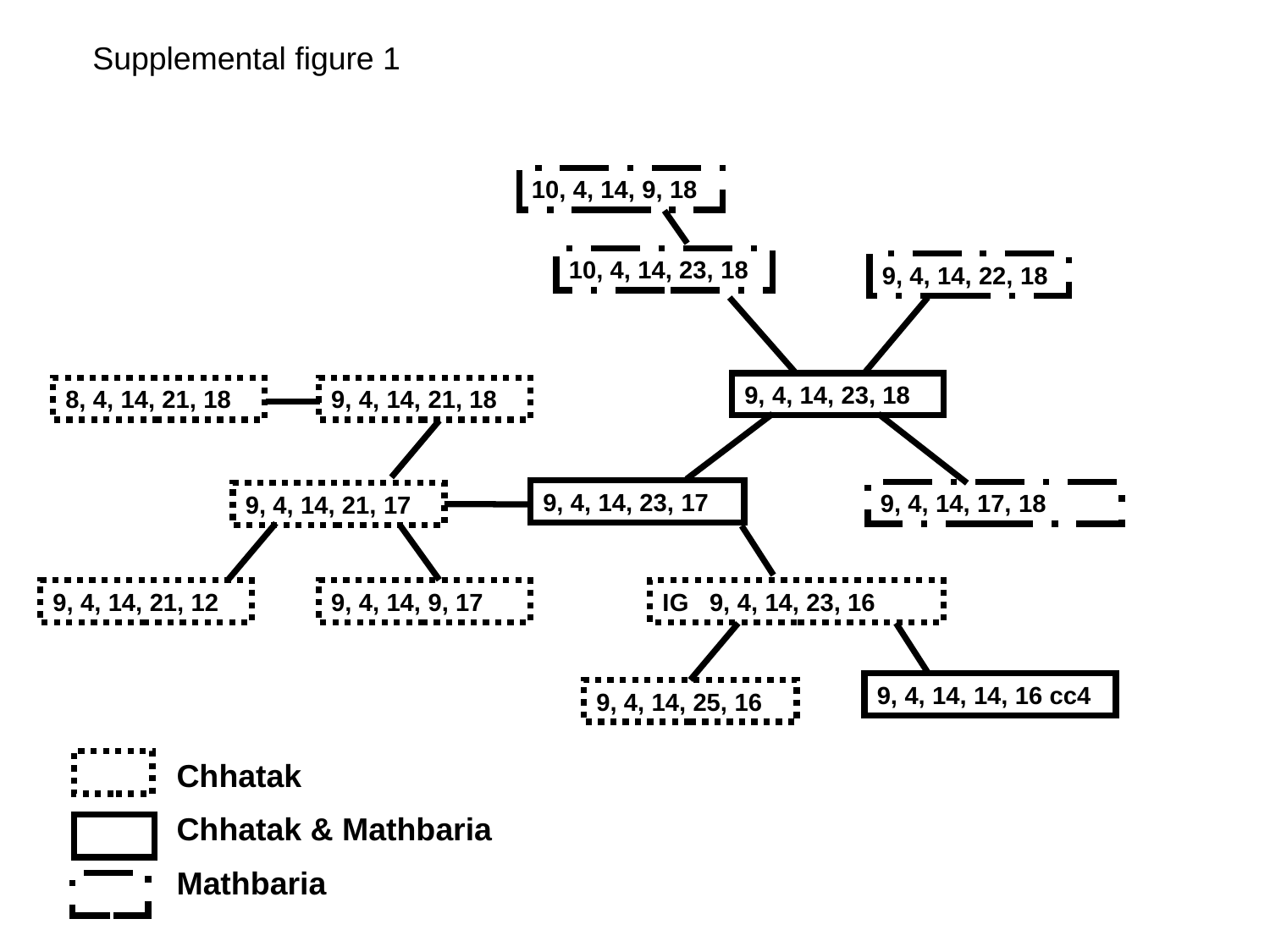

Supplemental figure 1
10, 4, 14, 9, 18
10, 4, 14, 23, 18
9, 4, 14, 22, 18
9, 4, 14, 23, 18
8, 4, 14, 21, 18
9, 4, 14, 21, 18
9, 4, 14, 23, 17
9, 4, 14, 17, 18
9, 4, 14, 21, 17
9, 4, 14, 21, 12
IG 9, 4, 14, 23, 16
9, 4, 14, 9, 17
9, 4, 14, 14, 16 cc4
9, 4, 14, 25, 16
Chhatak
Chhatak & Mathbaria
Mathbaria

Supplement: Supplementary Data [file fnw116_supplementary_data.zip › figure S1.pptx]

## Slide 1
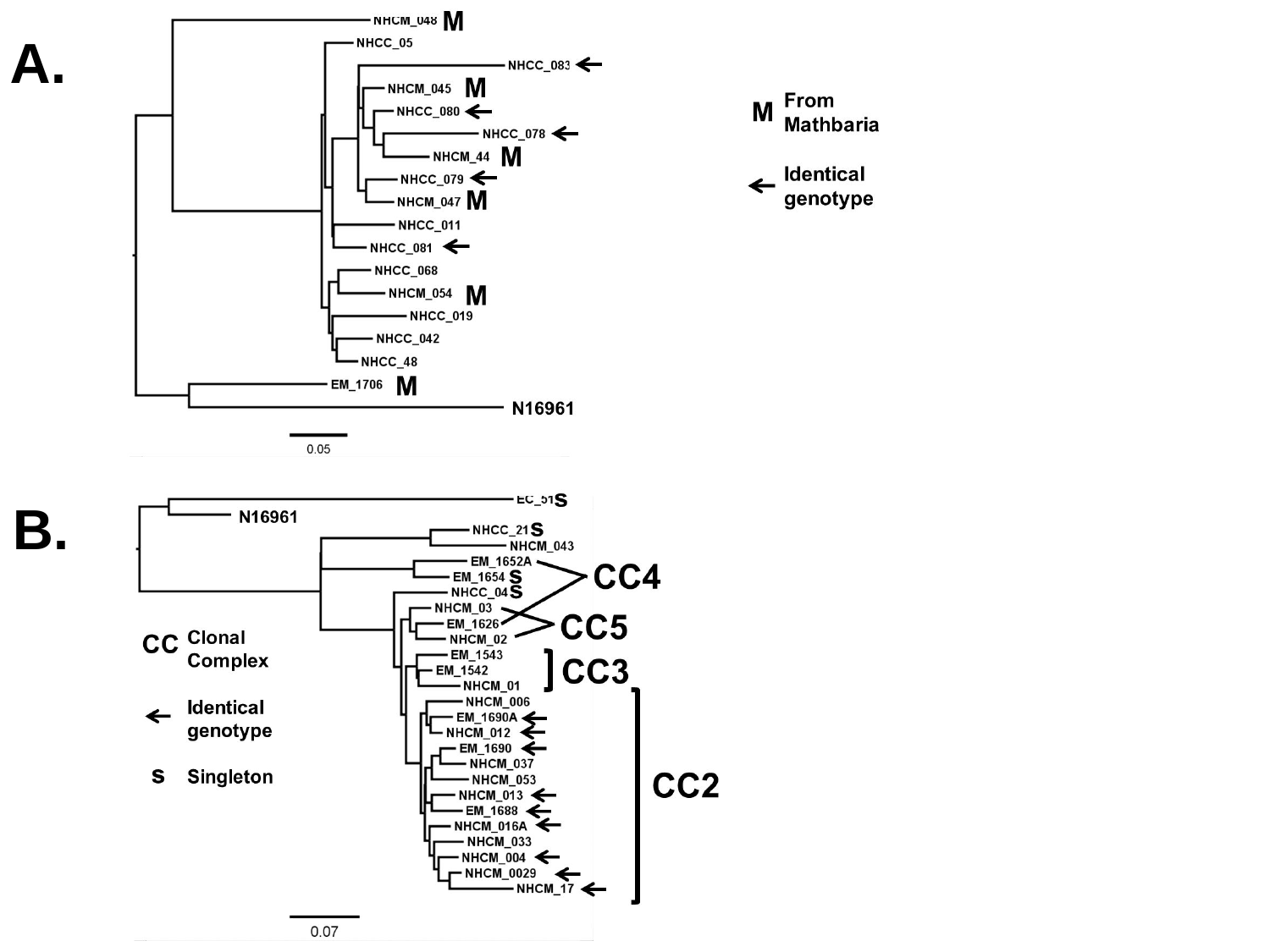

A.
B.

Supplement: Supplementary Data [file fnw116_supplementary_data.zip › figure S2.pptx]
