# Supplementary material for: Comparison of inferred relatedness based on multilocus variable-number tandem-repeat analysis and whole genome sequencing of Vibrio cholerae O1
Source: FEMS Microbiol Lett. 2016 Apr 28;363(12):fnw116. doi: 10.1093/femsle/fnw116 (PMC4876684; doi:10.1093/femsle/fnw116)
Supplement: Supplementary Data [file fnw116_supplementary_data.zip › Supplemental Figure Legend.doc]

**Supplemental Figure 1.** The five locus MLVA genotypes of the 42 isolates included in this study are shown in boxes. Lines between boxes represent an allelic change at a single locus. Genotypes of CC1 obtained both Chhatak and Mathbaria have solid lines, genotypes found only in Chhatak have dotted lines, genotypes only in Mathbaria lines are indicated with dashes and dots. The IG in one box stands for intermediate genotype. An isolate with this genotype was observed in Chhatak, but was not sequenced in this study. The cc4 in box identifies the genotype found in CC1 in Chhatak and CC4 in Mathbaria.

**Supplemental Figure 2.** Maximum likelihood phylograms of WGS data from isolates in MLVA. The arrows identify those isolates with the identical MLVA genotype. In a phylogram the horizontal length is proportional to the number of nucleotide changes. The terminal branches are longer than the stems. A) CC1. ‘M’ marks those isolates from Mathbaria, the others are from Chhatak. B) CC2, CC3, CC4, CC5 and the singletons.
